# Subclinical myocardial dysfunction is revealed by speckle tracking echocardiography in patients with Cornelia de Lange syndrome

**DOI:** 10.1007/s10554-022-02612-0

**Published:** 2022-05-19

**Authors:** Laura Trujillano, Ariadna Ayerza-Casas, Beatriz Puisac, Gonzalo González García, Ángela Ascaso, Ana Latorre-Pellicer, María Arnedo, Cristina Lucia-Campos, Marta Gil-Salvador, Frank J. Kaiser, Feliciano J. Ramos, Juan Pié, Gloria Bueno-Lozano

**Affiliations:** 1 Unit of Clinical Genetics and Functional Genomics, Department of Pharmacology-Physiology-Legal Medicine, School of Medicine, Universidad de Zaragoza, CIBERER-GCV02 and IIS-Aragon, Zaragoza, Spain; 2 Department of Clinical and Molecular Genetics Medicine Genetics Group, VHIR, University Hospital Vall d’Hebron, Barcelona, Spain; 3 Department of Pediatrics, Hospital Clínico Universitario “Lozano Blesa”, CIBERER-GCV02 and IIS-Aragon, Zaragoza, Spain; 4 Department of Pediatrics, Hospital Universitario Miguel Servet, Zaragoza, Spain; 5 Department of Pediatrics, Hospital Clínico Universitario Lozano Blesa, Zaragoza, Spain; 6 Institute for Human Genetics, University Hospital Essen, University of Duisburg-Essen, 45147 Essen, Germany; 7 Center for Rare Diseases (Essener Zentrum für Seltene Erkrankungen, EZSE), University Hospital Essen, Essen, Germany

**Keywords:** Echocardiography, Speckle tracking, Strain, Myocardial dysfunction, Cornelia de, Lange syndrome, NIPBL

## Abstract

This study assesses a possible cardiac dysfunction in individuals with Cornelia de Lange syndrome (CdLS) without diagnosed congenital heart disease (CHD) and its association with other factors. Twenty patients and 20 controls were included in the study divided into three age-dependent groups (A: < 10 yrs, B: 10–20 yrs, C: > 20 yrs), and were evaluated using conventional echocardiography, tissue doppler imaging (TDI), two-dimensional speckle tracking and genetic and biochemical analyses. The left ventricular global longitudinal strain (GLS) was altered (< 15.9%) in 55% of patients, being pathological in the older group (A: 19.7 ± 6.6; B: -17.2 ± 4.7; C: -13.6 ± 2.9). The speckle tracking technique revealed a downward trend in the values of strain, strain rate and velocity, especially in the oldest group. Likewise, the ejection fraction (LVEF) and shortening fraction (LVFS) values, although preserved, also showed a decreased with age (p < 0.05). The analytical markers of cardiovascular risk and cardiac function showed no alterations. The molecular analyses revealed 16 individuals carrying pathogenic variants in NIPBL, two with variants in SMC1A, one with a variant in RAD21 and one with a HDAC8 variant. This is the first systematic approach that demonstrates that individuals with CdLS may present early cardiomyopathy, which can be detected by speckle tracking technique even before the appearance of clinical symptoms and the alteration of other echocardiographic or analytical parameters. For all these reasons, cardiological followup is suggested even in the absence of CHD, especially from adolescence onwards.

## Introduction

Cornelia de Lange syndrome (CdLS) (OMIM #122,470, #300,590, #610,759, #614,701, #300,882) is a congenital multisystemic-malformation syndrome with an estimated incidence of one per 10,000–30,000 live births [1].

In the vast majority of patients, pathogenic variants occur de novo in genes encoding structural and regulatory components of the cohesin complex that, inter alia, are involved in the regulation of gene expression [2].

The most frequently observed genetic cause of CdLS is a heterozygous pathogenic variant in the *NIPBL* gene, which accounts for up to 70% of cases [3, 4]. However, deleterious variants in seven other genes are known to be responsible for this condition (*SMC1A, SMC3, RAD21* [5], *BRD4, HDAC8, ANKRD11* [6] and *MAU2* [7]). However, up to 10–20% of patients still remain undiagnosed, probably due to the existence of additional causal genes, somatic mosaicism [8, 9], and copy-number variants (CNVs) [10]. Currently, whole-exome sequencing (WES) and targeted panels of next-generation sequencing (NGS) identify variations in these genes and are the most effective tools for the molecular diagnosis of CdLS [11].

CdLS is characterized by typical facial features, growth failure, and limb abnormalities but also affects multiple organs and systems. In addition to the characteristic facial dysmorphism, growth retardation, intellectual disability, and gastroesophageal reflux, congenital heart disease (CHD) has been reported in a large number of CdLS patients [12]. Despite the numerous health conditions, most of CdLS patients will live through adulthood [1]. Congenital heart defects have been widely studied and are present in 30–40% of CdLS cases. Pulmonary stenosis and septal defects are the most common anomalies [13]. Despite the systemic involvement of the disease, there are few data on whether myocardial dysfunction may exist. However, acquired cardiac defects as well as cardiomyopathy or congestive heart failure are responsible for 3% of deaths [14–16].

In a previous study of cardiac function in patients with CdLS without CHD using conventional two-dimensional echocardiography, an age-related downward trend in left ventricular ejection fraction (LVEF) was observed. However, all values were in the normal range and all patients were asymptomatic [17]. Although the ejection fraction is the most commonly used parameter for the evaluation of systolic function, it has low sensitivity for the assessment of early changes in contractile function. In this sense, interest in strategies sensitive enough to detect early myocardiopathy is rising. Speckle tracking echocardiography is an advanced echocardiographic technique that allows quantification of myocardial deformation, also called strain, by using semiautomated software. This technique has strengthened the ability to assess left ventricular function. It is reportedly a sensitive marker for early subtle abnormalities of left ventricular myocardial performance. This makes it helpful for the prediction of outcomes for various cardiac diseases, including cardiomyopathy, and superior to conventional echocardiographic indices [18–20]. In addition, it has proven to be useful for the early detection of myocardial dysfunction of the left ventricle in children with different genetic conditions [21–23].

The aim of this study was to evaluate cardiac function in patients with CdLS without CHD by using classical techniques, such as conventional echocardiography, the analysis of biochemical markers, and the two-dimensional speckle tracking technique, and assess its possible correlations with other factors in cases of myocardial dysfunction.

## Materials and methods

### Patient cohort

This case–control study included 20 individuals with CdLS (6 males, 14 females, all Caucasian, aged 2–45 years) without CHD who were asymptomatic for cardiovascular disease and 20 healthy controls of the same age and sex. The sample was divided into three age groups (A: < 10, B: 10–20, and C: > 20 years). In addition, all individuals with CdLS were subjected to molecular analysis using WES or targeted NGS panels including the *NIPBL, SMC1A, SMC3, RAD21, HDAC8, ANKRD1, BRD4* and *MAU2* genes. All patients, or their parents or guardians, signed a written consent form to participate in this study, which was approved by the Ethics Committee of Clinical Research from the Government of Aragón (Spain) (CEICA; PI16/225).

### Anthropometric measurements and clinical records

Individuals with CdLS underwent anthropometric and physical examinations, and medical records were collected from their clinical histories. Weight was measured in kilograms (kg) using an AMGI-IMSA model, and height was measured in centimeters (cm) using the Harpenden model tallimeter. BMI was calculated by dividing weight (kg) by square height (m^2^). We categorized CdLS phenotypes using the consensus criteria recently published in the first international consensus statement [1]. Classic CdLS was indicated with a clinical score > 11 and at least three of the cardinal features, nonclassic CdLS was indicated with a score of 9–10 and two of the cardinal features, and a score of ≥ 4 was sufficient to justify molecular testing for CdLS if one cardinal feature was present [1]. Heart rate, systolic blood pressure (SBP) and diastolic blood pressure (DBP) were monitored in all patients.

### Biochemical markers

Blood samples were requested from CdLS patients (with the exception of four patients) to measure myocardial function biochemical markers (troponin-T and NT-ProBNP) and cardiovascular risk factors (total cholesterol, LDL-c, HDL-c and triglycerides) by standard analysis procedures.

### Echocardiographic examination

A Siemens ACUSON SC2000 ultrasound system was used for cardiac evaluation. All measurements were carried out by two different researchers to decrease the interobserver variability. Two-dimensional ultrasound, color Doppler and tissue Doppler measurements were performed according to the recommendations of the *American Society of Echocardiography* and the *European Association of Cardiovascular Imaging* [18, 24].

**Fig. 1:**
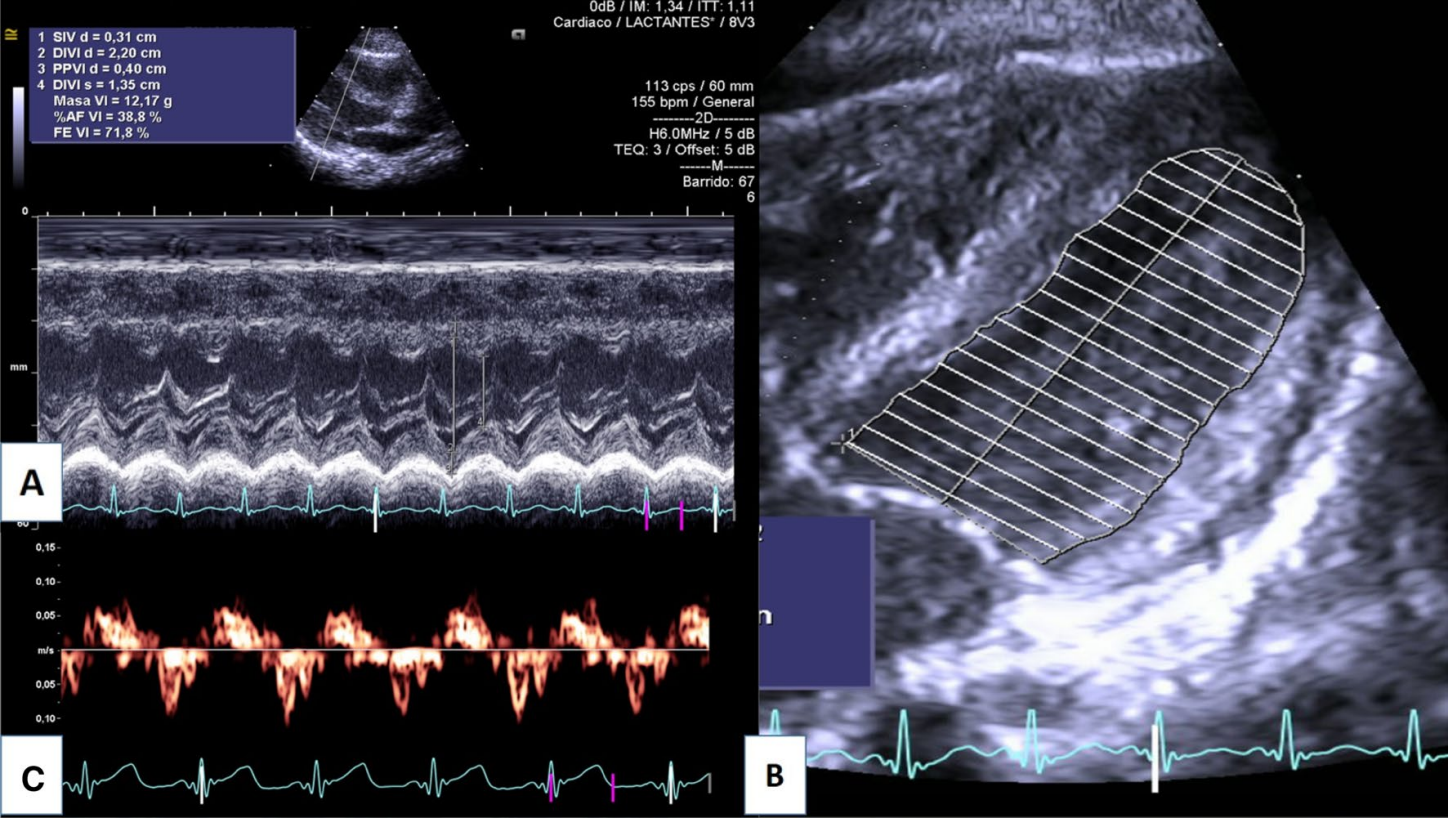
2D Echocardiography. **A** IVSd, interventricular septum thickness at end-diastole; LVIDd, left ventricular internal dimension at end-diastole; LVIDs, left ventricular internal dimension at end-systole; PWD, posterior wall at diastole; LVM, Left ventricular mass. **B** LVEF, left ventricular ejection fraction using biplane Simpson´s method. **C** Left ventricular tissue doppler velocities (s´,e´,a’) of the lateral side of the mitral annulus

We calculated the interventricular septum thickness at end-diastole (IVSd), left ventricular internal dimension at end-diastole (LVIDd), left ventricular internal dimension at end-systole (LVIDs), posterior wall at diastole (PWd), left ventricular mass in grams (LV mass) (Fig. 1A), right ventricular at end-diastole (RVD), left ventricular shortening fraction (LVFS) and tricuspid annular plane systolic excursion (TAPSE) using the M-mode method**.** The left ventricular ejection fraction (LVEF) was calculated using biplane Simpson’s method (Fig. 1B).

Mitral inflow Doppler velocities and the peak early (E-wave) and late filling (A-wave) were measured by using pulsed-wave Doppler after placing the sample volume at the leaflets’ tips.

Left ventricle tissue Doppler velocities, systolic velocity (s’), diastolic early (e’) and late (a’) lateral mitral annular velocities were calculated after placing the sample volume of the pulsed-wave Doppler at the lateral side of the mitral annulus (Fig. 1C). Left ventricular diastolic function was evaluated using the E/e’ ratio, lateral e’, tricuspid regurgitation velocity, left atrial volume index and E/A ratio [24].

### Speckle tracking echocardiography

**Fig. 2:**
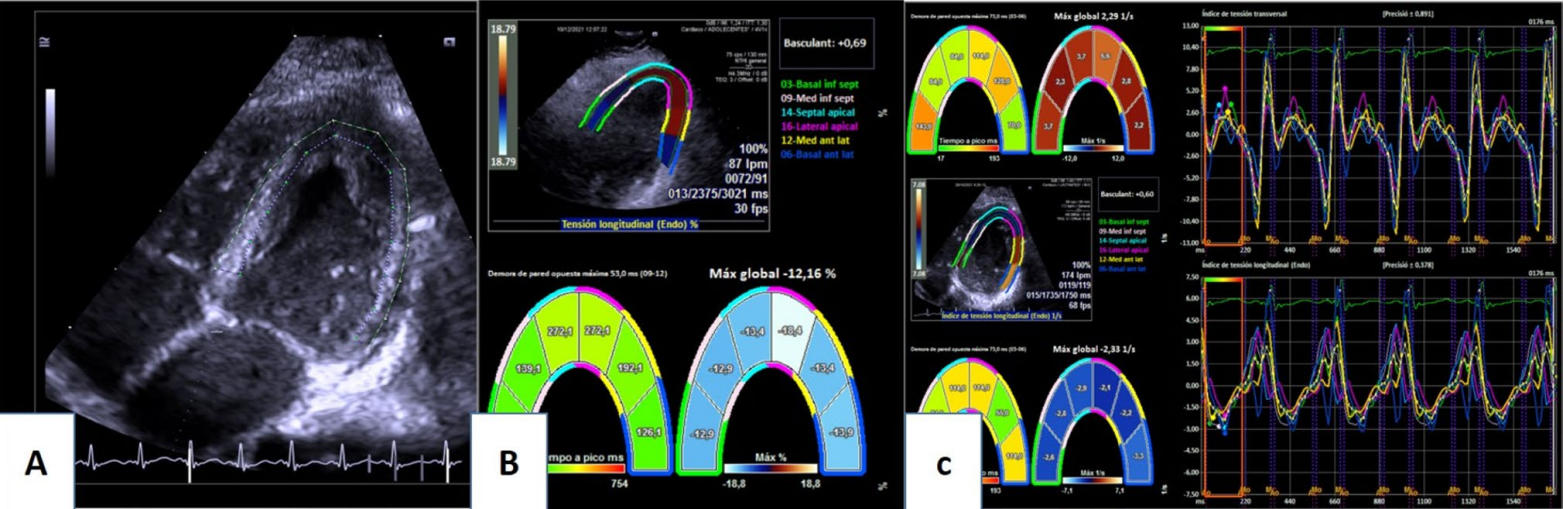
Speckle tracking echocardiography. **A** Four chambers plane for speckle tracking calculation. **B** Left ventricular global strain (%). **C** Strain rate of left ventricle (1/s)

The images were acquired using the same echocardiography equipment, and Velocity Vector Imaging 3.0 software was used to obtain left ventricular global longitudinal strain (GLS-LV) measurements. The cardiac cycle at end-diastole was selected on the echocardiogram, coinciding with the onset of the QRS complex. The four chamber planes of three cardiac cycles were acquired with a comprehensive adjustment of the image quality and a temporal resolution ≥ 60 frames/s (Fig. 2A). In the case of an inadequate trace, the affected segment was excluded from the analysis, allowing a maximum of two of six segments eliminated to consider the study valid. Left ventricular global longitudinal strain (%) (Fig. 2B), strain rate (1/s) (Fig. 2C) and velocity (cm/s) measurements were obtained.

Global longitudinal strain and strain rate values are expressed in absolute values. Reference values according to the different meta-analyses for global longitudinal strain vary between 15.9% and 22.1%, with a mean of 19.7% and a 95% confidence interval [25–30]. We categorized strain values below 15.9% as abnormal.

### Statistical analysis

Data are presented as mean (standard deviation) values and percentages. The comparison between patients with CdLS and the control group was made using the Mann–Whitney or chi-square tests. A correlation study was performed by calculating Pearson’s correlation coefficient and scatter plots. A p value lower than 0.05 was considered statistically significant. Statistical analyses were performed using IBM SPSS Statistics® 20.0. software, and graphics were produced with GraphPad Prism 8 software.

## Results

Somatometric measurements of the individuals with CdLS and individuals in the control group are shown in Table 1. The clinical score was calculated in 18 of 20 patients. Thirteen of the 18 individuals presented a classic phenotype with a score ≥ 11, two presented a nonclassical phenotype with a score between 9 and 10, and the remaining three obtained a score between 4 and 8 (Table 2). Molecular analyses revealed that 16 individuals carried a pathogenic variant in the *NIPBL* gene, two carried a pathogenic variant in *SMC1A*, one carried a pathogenic variant in *RAD21* and one carried a pathogenic variant in *HDAC8* (Table 2).

**Table 1:** Characteristics of our cohort

Variables	CdLS group (n = 20)	Control group (n = 20)
Gender (female/male)	6/14	6/14
Age (y)	13.9 ± 11	13.9 ± 10.6
Weight (kg)	29.5 ± 21.8	39.2 ± 18.6
Heigh (cm)	120.3 ± 26.9	139.7 ± 28.4
BSA (m^2^)	0.9 ± 0.4	1.2 ± 0.4

*BSA* Body Surface Area

*Groups were compared by Mann–Whitney U test

**Table 2:** Characteristics of CdLS individuals and controls

	I1	I2	I3	I4	I5	I6	I7	I8	I9	I10	I11
Age (years)	2.1	2.5	3.5	3.7	5.2	5.5	5.5	8.5	9.9	11	11.5
Gender	M	M	F	M	F	F	F	F	F	F	F
Gene	*HDC8*	*NIPBL*	*NIPBL*	*NIPBL*	*RAD21*	*SMC1A*	*NIPBL*	*NIPBL*	*NIPBL*	*NIPBL*	*SMC1A*
Clinical score	11	14	15	14	8	5	9	13	–	14	14
Strain (%)	13.4	31.2	14.2	16.6	29.9	15.5	20.1	15.4	21.2	15.7	20.5
Strain rate (1/s)	1.5	2.1	1.1	1.3	1.1	1.1	0.1	0.7	1.7	1.5	2.3
Velocity (cm/s)	1.4	3.3	2.2	1.8	1.5	2.9	1.7	2.3	1.4	1.6	1.1
	I12	I13	I14	I15	I16	I17	I18	I19	I20		
Age (years)	14	15.5	16,9	17.5	20.8	23.5	25	31	45		
Gender	M	F	F	M	F	M	F	F	F		
Gene	*NIPBL*	*NIPBL*	*NIPBL*	*NIPBL*	*NIPBL*	NIPBL	*NIPBL*	*NIPBL*	*NIPBL*		
Clinical score	15	7	15	14	16	13	–	9	15		
Strain (%)	23.4	10.5	14	19.3	13.8	13.7	18.2	11	11.4		
Strain rate (1/s)	1	1	0.9	1.2	1.3	1.2	1.5	0.5	0.6		
Velocity (cm/s)	2.9	2.1	2.1	2.1	2	2.4	2.7	1.3	0.9		
	C1	C2	C3	C4	C5	C6	C7	C8	C9	C10	C11
Age (years)	2	2.7	4	5	5.5	6	6.8	9	9	10.1	10.2
Gender	M	M	F	M	F	F	F	F	F	F	F
Strain (%)	19.0	18.7	25.5	20.8	19.7	22.1	18.4	23.3	19.6	19.7	23.9
Strain rate (1/s)	2	1.4	2	1.9	1.3	1.6	1.9	1.8	1.6	1.7	1.9
Velocity (cm/s)	1.6	2.4	3.5	3.3	2.7	2.6	2.9	2.8	3.4	3.3	3.3
	C12	C13	C14	C15	C16	C17	C18	C19	C20		
Age (years)	13	14	17	18	21	25	27	29	43		
Gender	M	F	F	M	F	M	F	F	F		
Strain (%)	20.7	23.9	19.6	20.5	18.8	20.2	18.1	19.1	27.2		
Strain rate (1/s)	2	1.7	2.2	2.4	1.9	1.1	1.3	1.4	2.3		
Velocity (cm/s)	3.7	4.1	2.1	2	1.8	3.3	3.3	3.3	3.4		

Strain (%) and Strain rate are expressed in absolute values

Systolic and diastolic blood pressure values were within the range considered normal in almost all age groups but were significantly higher in individuals with CdLS from the A group (SBP A: 112 ± 20 vs. 96 ± 5 mm Hg, p = 0.04; B: 114 ± 18 vs. 111 ± 6 mm Hg, p = 0.132; C: 105 ± 22 vs. 112 ± 3 mm Hg, p = 0.286 and DBP A: 69 ± 10 vs. 55 ± 4 mm Hg, p = 0.005; B: 69 ± 13 vs. 64 ± 5 mm Hg, p = 0.122; C: 71 ± 20 vs. 68 ± 5 mm Hg, p = 0.056).

The analytical markers of myocardial function (troponin-T and NT-ProBNP) and cardiovascular risk (total cholesterol, LDL-c, HDL-c and triglycerides) were within the normal range in all cases.

**Fig. 3:**
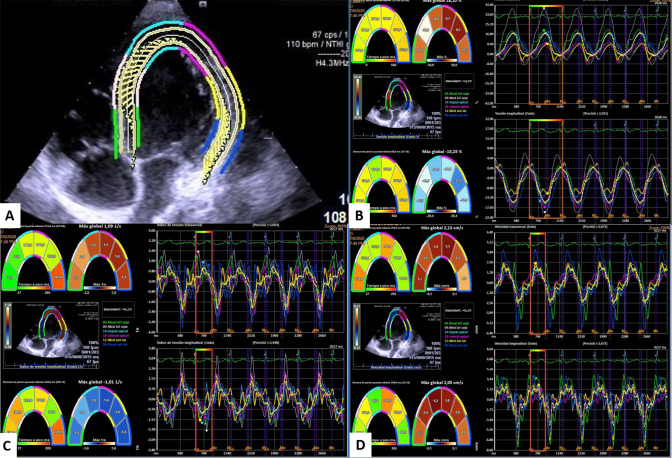
Image obtained by speckle tracking in SCdL individual

**Fig. 4:**
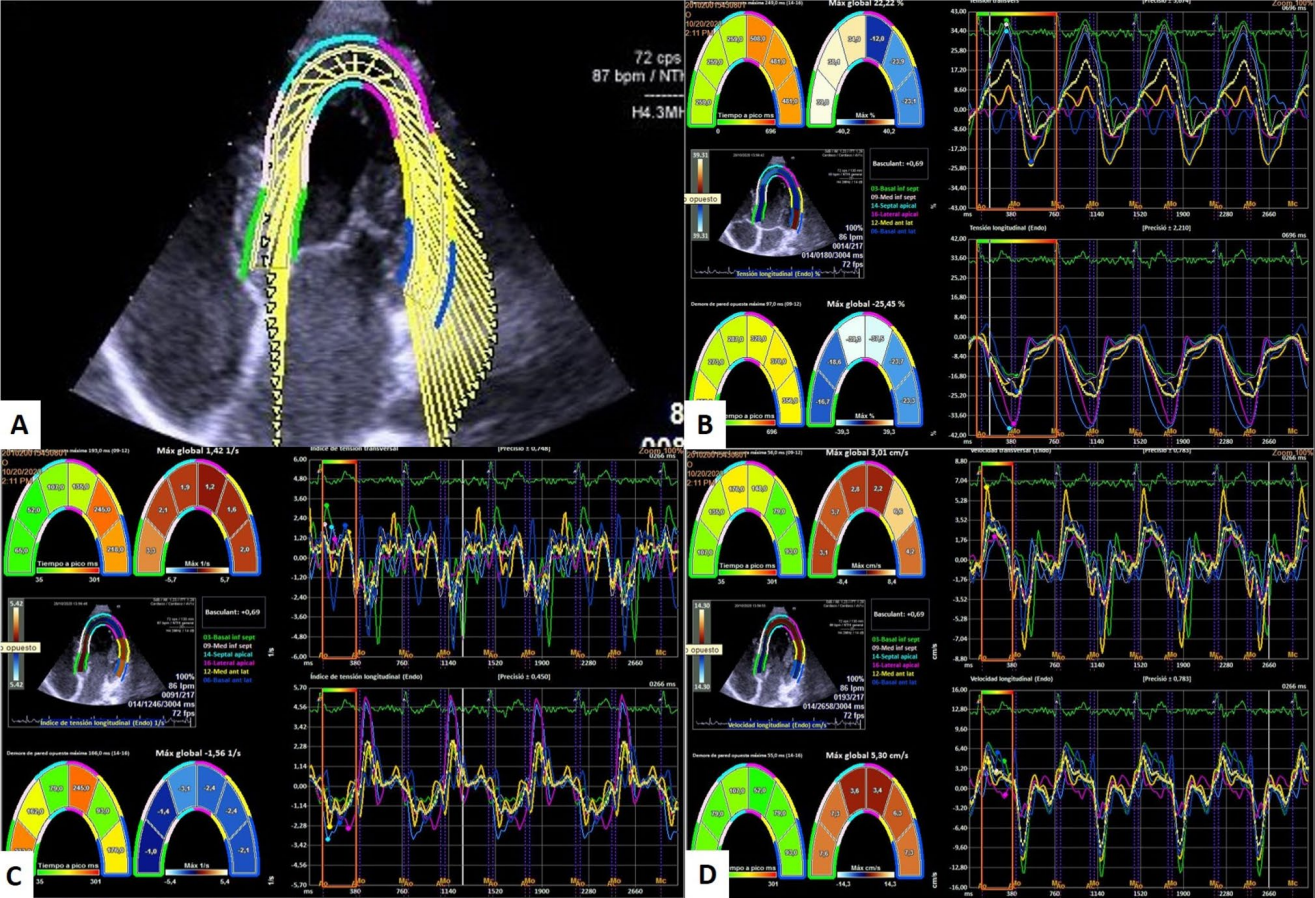
Image obtained by speckle tracking in a healthy control

The echocardiographic values obtained after comparison of the control and study groups are shown in Table 3. Left ventricular mass (g/m2) and TAPSE (mm) values were significantly decreased in individuals with CdLS, as were the absolute value of strain (GLS-LV), strain rate and velocity values. This study (Table 2) revealed that 55% (11/20) of patients presented an abnormal left ventricular global longitudinal strain (GLS-LV < 15.9%) (Table 2). Figure 3 shows the image obtained after performing speckle tracking in a CdLS patient, presenting clearly decreased values compared to those obtained in a healthy control individual (Fig. 4).

**Table 3:** Comparison of echocardiography findings from CdLS individuals and unaffected controls

Variables	CdLS (n = 20)	Control (n = 20)	p value
IVSd (mm)	6.4 ± 1.8	7 ± 1.1	0.123
LVIDd (mm/m^2^)	38.9 ± 13.8	35.8 ± 9.2	0.705
LVIDs (mm/m^2^)	22.6 ± 7.1	21.2 ± 5.1	0.534
PWD (mm)	6.8 ± 2.1	6.8 ± 1.3	0.850
LVM (g/m^2^)	53.5 ± 15.4	63.6 ± 12.1	**0.030**
RVD (mm)	18.5 ± 5	16.8 ± 4.1	0.250
LVEF (%)	68.8 ± 6.3	67.9 ± 3.9	0.871
LVFS (%)	40.9 ± 5.9	41.4 ± 3.9	0.755
TAPSE (mm)	18.9 ± 2.9	21.4 ± 3.5	**0.020**
E wave (cm/s)	10 ± 2	10 ± 1	0.607
A wave (cm/s)	6 ± 1	5 ± 1	0.198
E/A	1.8 ± 0.5	2 ± 0.3	0.104
TDI “s” wave (cm/s)	11 ± 2	10 ± 2	0.233
TDI “e” wave (cm/s)	16 ± 4	16 ± 3	0.654
TDI “a” wave (cm/s)	7 ± 2	7 ± 1	0.397
E/e’	6.6 ± 1.6	6.6 ± 1.2	0.490
GLS-LV (%)	17.5 ± 5.7	20.9 ± 2.5	**0.006**
Strain rate (1/s)	1.2 ± 0.4	1.8 ± 0.3	** < 0.001**
Velocity (cm/s)	2 ± 0.6	2.9 ± 0.7	** < 0.001**

*IVSd* interventricular septum thickness at end-diastole, *LVIDd* left ventricular internal dimension at end-diastole, *LVIDs* left ventricular internal dimension at end-systole, *PWD* posterior wall at diastole, *LVM* Left ventricular mass in grams, *RVD* Right ventricular diameter, *LVEF* left ventricular ejection fraction, *LVFS* left ventricular fractional shortening, *TAPSE* tricuspid annular plane systolic excursion, *TDI* lateral mitral tissue Doppler imaging, *GLS-LV* Global longitudinal strain for left ventricular function

*GLS-LV and Strain rate are expressed in absolute values.* Groups were compared by Mann–Whitney U test; statistically significant results are highlighted in bold

The diastolic function parameters are shown in Table 3. In all individuals, the tricuspid regurgitation velocity was below 2.8 m/s, and the left atrial index volume was below 34 mL/m2.

When searching for correlations between the presence of this subclinical myocardial dysfunction and other factors (age, sex, anthropometric data, clinical score, biochemical markers, molecular markers, and echocardiographic markers), age was the only feature that seemed to correlate.

**Fig. 5:**
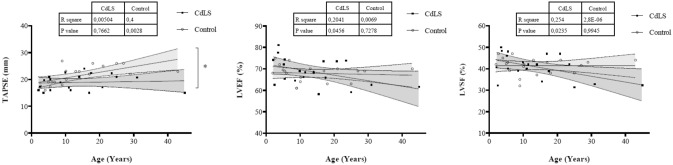
Evolution of TAPSE (%), LVFE (%) and LVSF (%) in CdLS compared to the control group

It is worth noting that when the cohort of the study was divided into different age groups, a decrease in speckle tracking values was observed in all patient groups compared to appropriate controls. This was especially significant in the oldest group. The remaining values resulting from conventional echocardiography are shown in Table 4. When we correlated these values with age, a downward ejection fraction (LVEF) and shortening (LVFS) (p = 0.046 and p = 0.023) were observed. However, all of these values were within the normal range (Fig. 5).

**Table 4:** Comparison of echocardiographic findings from CdLS individuals and unaffected controls by age group

Age group	0 – 10 years			10 – 20 years			> 20 years		
	CdLS (n = 9)	Control (n = 9)	*p*	CdLS (n = 6)	Control (n = 6)	*p*	CdLS (n = 5)	Control (n = 5)	*p*
IVSd (mm)	5.2 ± 0.9	6.2 ± 0.6	**0.024**	6.7 ± 1.3	8 ± 0.9	0.065	8.2 ± 2	7.4 ± 0.7	0.251
LVIDd (mm/m^2^)	50.4 ± 11.8	44.5 ± 7	0.270	30.3 ± 7.3	28.6 ± 2.1	0.749	28.7 ± 3.4	28.7 ± 1.9	0.602
LVIDs (mm/m^2^)	28.4 ± 6.1	26.2 ± 2.5	0.453	18 ± 4.1	17.4 ± 2.02	0.631	17.7 ± 1.4	16.6 ± 1.3	0.076
PWD (mm)	5.5 ± 1	5.8 ± 0.8	0.450	8.4 ± 2.7	7.3 ± 1.2	0.297	7.5 ± 1.1	7.8 ± 0.9	0.530
LVM (g/m^2^)	51.7 ± 16	60.4 ± 8.7	0.270	50.7 ± 13.6	63 ± 15.6	0.337	60 ± 17.5	70.1 ± 12.9	0.117
RVD (mm)	15.7 ± 2.8	14.1 ± 2	0.156	18.7 ± 3.5	16.8 ± 4.8	0.522	23.2 ± 6.5	21.4 ± 1.1	0.602
LVEF (%)	71.2 ± 6.2	68.6 ± 5	0.331	66.8 ± 5	66.7 ± 2.8	0.873	66.2 ± 6.9	68.2 ± 2.9	0.599
LVFS (%)	43 ± 5.7	41.4 ± 5.2	0.479	40.7 ± 4.3	40.9 ± 2.8	0.872	37.1 ± 7	42.1 ± 2.5	0.249
TAPSE (mm)	18.6 ± 2.6	18.7 ± 3.3	0.965	19.2 ± 3.7	22.9 ± 1.9	0.109	19.2 ± 3	24.4 ± 1.8	**0.016**
E wave (cm/s)	11 ± 2	11 ± 2	0.859	1 ± 1	1	0.872	8 ± 1	10 ± 1	0.116
A wave (cm/s)	6 ± 1	6 ± 1	0.790	6 ± 1	5 ± 1	0.092	5 ± 1	5 ± 1	0.834
E/A	2 ± 0.6	1.9 ± 0.3	0.965	1.7 ± 0.2	2 ± 0.3	0.054	1.6 ± 0.3	1.9 ± 0.3	0.116
TDI “s” wave (cm/s)	11 ± 2	9 ± 2	0.101	11 ± 2	10 ± 1	0.810	11 ± 1	12 ± 2	0.834
TDI “e” wave (cm/s)	19 ± 4	15 ± 2	**0.046**	15 ± 3	17 ± 3	0.053	14 ± 4	18 ± 2	**0.028**
TDI “a” wave (cm/s)	7 ± 1	7 ± 1	1	7 ± 1	7 ± 1	0.514	7 ± 4	6 ± 1	0.600
E/e’	6.3 ± 1.6	7.3 ± 1	**0.046**	7.4 ± 1.8	6.2 ± 0.9	0.262	6.1 ± 1.3	5.8 ± 1	0.917
GLS-LV (%)	19.7 ± 6.6	20.8 ± 2.4	0.310	17.2 ± 4.7	21.4 ± 2	0.078	13.6 ± 2.9	20.7 ± 3.7	**0.016**
Strain rate (1/s)	1.3 ± 0.4	1.7 ± 0.2	**0.024**	1.3 ± 0.5	2 ± 0.3	**0.037**	1 ± 0.5	1.6 ± 0.5	0.175
Velocity (cm/s)	2 ± 0.7	2.8 ± 0.6	**0.038**	2 ± 0.6	3.1 ± 0.8	**0.045**	1.9 ± 0.8	3 ± 0.7	**0.047**

*IVSd* interventricular septum thickness at end-diastole, *LVIDd* left ventricular internal dimension at end-diastole, *LVIDs* left ventricular internal dimension at end-systole, *PWD* posterior wall at diastole, *LVM* Left ventricular mass in grams, *RVD* Right ventricular diameter, *LVEF* left ventricular ejection fraction, *LVFS* left ventricular fractional shortening, *TAPSE* tricuspid annular plane systolic excursion, *TDI* lateral mitral tissue Doppler imaging, *GLS-LV* Global longitudinal strain for left ventricular function. GLS-LV and Strain rate are expressed in absolute values

Groups were compared by Mann–Whitney U test; statistically significant results are highlighted in bold

**Fig. 6:**
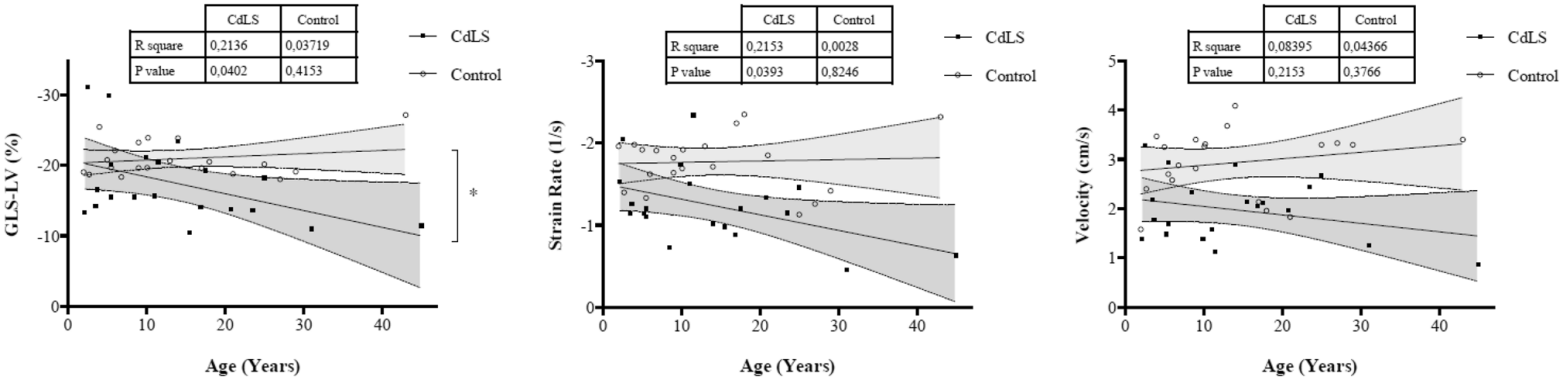
Evolution of Global Longitudinal Strain (%), Strain Rate (1/s) and velocity (cm/s) in CdLS compared to the control group

The lateral e’ wave values of the mitral annulus showed significantly lower values in individuals with CdLS over the age of 20. The speckle tracking technique also revealed a downward trend in the values of strain (GLS-LV), strain rate and velocity, resulting abnormal in most cases (Fig. 6).

## Discussion

Cardiovascular defects represent a significant cause of morbidity and mortality in patients with CdLS. CHD has been described in one-third of the total individuals affected. The most frequent CHDs are septal defects (50%), pulmonary stenosis (27%) and coarctation of the aorta (9.6%). CHD contributes to approximately 25% of deaths during the first year of life [15]. Therefore, a thorough cardiological evaluation is of utmost importance for patients with a clinical diagnosis of CdLS [1].

There is limited information about the functional heart problems that may appear in these individuals, possibly because many of them did not reach adulthood in the past. However, a better knowledge of the molecular basis and, especially, the great advances that have been achieved in the clinical management of the syndrome have made it possible to extend their life expectancy. In the last few years, cases of heart failure and acquired cardiomyopathy have been described as responsible for 3% of deaths in patients with CdLS [31]. In a previous study, six patients with CdLS without CHD were analyzed for two years, and a decrease in LVEF was observed, although all values were within the normal range [15]. Similar results were obtained in a zebrafish model for cohesinopathies, in which a decrease in LVEF was observed [32]. In addition, experimental studies in Nipbl-mutated mice revealed alterations in the myocardium, presenting abnormal lacunar structures and disorganization of the compact layer [33]. Nevertheless, few studies have focused on the evolution of cardiac function in patients with CdLS.

Echocardiographic methods based on the LVEF parameter as an indicator of systolic function offer volumetric measurements that provide guidance on cardiac function. However, the speckle tracking technique allows us to determine the degree of myocardial fiber deformation (strain) and obtain more accurate measurements of systolic function. This may significantly aid in the detection of early myocardial damage. Several studies have already used this technique in clinical practice to assess cardiomyopathy, and they observed that strain and SR values are sometimes decreased when LVEF and other echocardiographic parameters are not even altered [34–36].

In the present study, the values obtained in individuals with CdLS with conventional echocardiography showed a downward trend in LVEF and LVFS, which correlated significantly with age. In addition, all values were within the normal range, suggesting that a cross-sectional echocardiographic analysis would not detect myocardial damage. In the same way, the TAPSE measurements, which correlate with the ejection fraction of the right ventricle, remained within the normal range. However, the mean values ​​were lower compared to controls, especially in the oldest group. These data agree with those previously published [17].

Although none of the individuals presented diastolic dysfunction [24], the mitral e-wave of tissue Doppler appears to be gradually reduced in elderly patients with CdLS (p < 0.05). This, together with the fact that the strain and strain rate are lower, would also support the presence of a certain degree of diastolic dysfunction. Moreover, early diastolic strain rate (SRd) values could add sensitivity and specificity to the conventional method, although a number of studies in childhood and adolescence are currently lacking [37].

In this sense, numerous studies support the usefulness of speckle tracking echocardiography for the evaluation of early systolic dysfunction. In fact, our study shows abnormal strain values in more than half of the patients, and their downward trend correlated with age. This technique could substantially aid in the early detection of cardiomyopathy [38].

In other genetic diseases, such as Duchenne muscular dystrophy, there is also a progressive decrease in strain values that allows early treatments. In individuals with Down syndrome, a decrease in strain values with preserved LVEF and increased left ventricular mass has been described [22, 23], but this has not been observed thus far in individuals with CdLS.

Although there is evidence that pathogenic variants in *NIPBL* and other genes of the cohesin complex alter heart development and produce structural defects, the underlying mechanisms of the development of cardiomyopathy in individuals without congenital malformations remain unknown. It has been proposed that patients with CdLS could undergo accelerated aging due to increased oxidative stress. This could prematurely deteriorate the heart, as it is an organ that is metabolically very active [39, 40].

Regarding phenotypic features, no differences were found their associations with the causal gene, although most of the patients in this study carried a pathogenic variant in the *NIPBL* gene.

Analytical markers of cardiovascular risk (triglycerides, total cholesterol, LDL cholesterol and HDL cholesterol) and myocardial function (ultrasensitive troponin T and N-terminal pro-brain natriuretic (NT-proBNP)) were within the normal range in all individuals studied. Although these biochemical parameters may be appropriate to identify cardiac dysfunction, they would not be useful for the early detection of these defects in patients with CdLS.

It is worth noting that CdLS is a rare genetic condition, and few individuals reach adulthood due to the progression of the disease. This is a significant limitation in this work, as the available sample size is small.

The results of this study suggest that patients with CdLS may develop subclinical cardiomyopathy, especially from the third decade of life onward. Taking this into account, it would be advisable to perform periodic cardiological evaluations that include the speckle tracking technique, despite having ruled out the existence of CHD in childhood.

The implementation of this in clinical practice for better follow-up of patients with CdLS would allow early medical treatment that might delay the development of the clinical manifestation of myocardial dysfunction.
